# Interaction of Adipocyte Metabolic and Immune Functions Through TBK1

**DOI:** 10.3389/fimmu.2020.592949

**Published:** 2020-10-20

**Authors:** Peng Zhao, Alan R. Saltiel

**Affiliations:** ^1^ Department of Medicine, University of California San Diego, La Jolla, CA, United States; ^2^ Department of Pharmacology, University of California San Diego, La Jolla, CA, United States

**Keywords:** TBK1, IKK, inflammation, metabolism, obesity, adipose tissue, overnutrition, undernutrition

## Abstract

Adipocytes and adipose tissue play critical roles in the regulation of metabolic homeostasis. In obesity and obesity-associated metabolic diseases, immune cells infiltrate into adipose tissues. Interaction between adipocytes and immune cells re-shapes both metabolic and immune properties of adipose tissue and dramatically changes metabolic set points. Both the expression and activity of the non-canonical IKK family member TBK1 are induced in adipose tissues during diet-induced obesity. TBK1 plays important roles in the regulation of both metabolism and inflammation in adipose tissue and thus affects glucose and energy metabolism. Here we review the regulation and functions of TBK1 and the molecular mechanisms by which TBK1 regulates both metabolism and inflammation in adipose tissue. Finally, we discuss the potential of a TBK1/IKK*ε* inhibitor as a new therapy for metabolic diseases.

## Introduction

Obesity has reached a pandemic (1). The complications of obesity, including type 2 diabetes, cardiovascular diseases, neurodegenerative diseases, non-alcoholic fatty liver diseases, and cancer, have become leading health threats. Obesity is caused by a positive energy balance, leading to excess lipid accumulation in adipose and other tissues (2–5). In addition to being an inert site for energy storage, adipose tissues play essential roles in metabolic homeostasis (6, 7). As the major cell type within adipose tissue, adipocytes are responsible for lipid storage and mobilization in response to insulin and sympathetic activation respectively. However, these cells can also sense their nutrient status, and respond by secreting a series of hormones known as “adipokines” (6–8). Upon food intake, the resulting elevation of nutrients in the circulation stimulates insulin production. Insulin in turn lowers glucose and fatty acid levels in part by instructing fat and muscle tissue to increase glucose uptake and storage, while reducing lipolysis in fat, glycogenolysis in muscle and liver and gluconeogenesis in liver (9, 10). In adipocytes, nutrients are largely stored as triglycerides. Upon reaching a threshold of lipogenesis, adipocytes trigger the production of adipokines such as leptin, to suppress food consumption and activate the sympathetic nervous system, thus closing a loop to ensure energy homeostasis (9, 11–15). Excessive energy intake or low energy expenditure could lead to a sustained positive energy balance and consequently cause increased adiposity in obesity (3–5).

Obesity is associated with low-grade chronic inflammation in adipose tissue, featured by an increased number of macrophages and an elevated ratio of proinflammatory macrophages (16–20). Although the immediate trigger for obesity-associated inflammation in adipose tissue remains unclear, multiple factors, including hypoxia, mechanical stress, lipotoxicity, adipocyte death, and bacterial toxins may contribute to this process (9, 21–27). Inflammation has been reported to affect several properties of adipocytes. The activation of proinflammatory pathways has been shown to disrupt glucose uptake and insulin responsiveness and alter adipokine production (28–31), suggesting that inflammation plays an essential role in the pathological response to obesity.

The nuclear factor kappa B (NF*κ*B) is a widely expressed transcription factor that mediates inflammatory responses in numerous tissues. The NF*κ*B signaling pathway plays a key role in the development of inflammation and insulin resistance in adipose tissue (32–34). Transcription through NF*κ*B is mainly controlled by the phosphorylation of inhibitor of NF*κ*B (I*κ*B) by the upstream I*κ*B kinases (IKKs). The canonical IKKs, IKK*α*, and IKK*β*, phosphorylate I*κ*B, and other NF*κ*B subunits to induce the expression of NF*κ*B target genes (35). Besides IKK*α* and *β*, the IKK family also includes two non-canonical members, IKK*ϵ* and TANK-binding kinase 1 (TBK1). Interestingly, despite their sequence similarity to the canonical IKK isoforms, TBK1 and IKK*ε* do not appear to play important roles in NFκB activation in response to proinflammatory cytokines (36). However, expression of *Ikke* and *Tbk1* mRNAs are induced by NF*κ*B (37). Moreover, IKK*ϵ* and TBK1 are activated by protein phosphorylation in response to proinflammatory cytokines or other substances that bind to Toll-like receptors 3 and 4 (38). It was reported that activities of IKK*ϵ* and TBK1 are significantly increased in adipose tissue of obese mice (37). We review here the functions of the noncanonical IKKs in inflammation and metabolic regulation in adipose tissue, with a major focus on the roles of TBK1 in crosstalk between inflammation and metabolism.

## Non-Canonical IKKs

NF*κ*B plays a central role in the transcriptional response to proinflammatory stimuli. In the absence of stimuli, I*κ*B binds to NF*κ*B to sequester the transcription factor in the cytoplasm (39). Inflammatory stimuli increase the phosphorylation and activation of IKKs, which in turn phosphorylate I*κ*B and NF*κ*B to activate the expression of NF*κ*B target genes (35, 39, 40). An IKK complex formed by IKK*α*, IKK*β*, and the NF*κ*B essential modifier (NEMO) directly phosphorylates I*κ*B at Ser^32^ and Ser^36^ to induce ubiquitin-associated degradation. Consequently, NF*κ*B is released to activate gene expression. This pathway represents the canonical NF*κ*B signaling pathway (41, 42). Both IKK*α* and IKK*β* possess a kinase domain (KD), a scaffold dimerization domain (SDD), and a NEMO-binding domain (NBD). A ubiquitin-like domain (ULD) is found in IKK*β* but not in IKK*α*. In contrast to the canonical IKKs, IKK*ϵ* and TBK1 have similar SD, ULD, and SDD, but lack the NBD. Human TBK1 shares 49% identity and 65% similarity to IKK*ϵ*, but only 27% identity with IKK*α* and IKK*β* (43–45). Unlike the canonical IKKs, the roles of IKK*ϵ* and TBK1 in the NF*κ*B signaling pathways remain uncertain. Early studies demonstrated that TBK1 phosphorylates IKK*β* to increase its activity, while IKK*ϵ* phosphorylates RelA at Ser^468^ to induce its nuclear translocation (44, 46, 47). However, subsequent studies found that TBK1 or IKK*ϵ* deficiency has no effect on LPS, TNFα, interleukin-1β, or poly(I:C)-induced activation of NF*κ*B (38, 48). Thus, it appears that IKK*ϵ* and TBK1 are not required for the activation of NF*κ*B in response to proinflammatory cytokines (36). Instead, studies showed that the expression of *Ikke* and *Tbk1* are induced by NF*κ*B under proinflammatory conditions (37). Interestingly, two separate studies demonstrated that TBK1 and IKK*ϵ* mediate NF*κ*B activation downstream of the cGAS-STING pathway in response to cytosolic DNA or STING ligand (49, 50).

Multiple studies demonstrated that non-canonical IKKs play important roles in metabolic regulation. The expression of *Ikke* was upregulated in the liver, adipocytes, and adipose tissue macrophages during diet-induced obesity (34). Knockout of *Ikke* reduced inflammation and improved insulin sensitivity in adipose tissue and liver. Hepatic steatosis was largely attenuated by IKK*ϵ* deficiency as well. *Ikke* knockout mice gained less weight and were resistant to high fat diet-induced obesity due to the increased energy expenditure and thermogenesis (34). The expression of Uncoupling protein 1 (*Ucp1*), a major uncoupler utilizing the mitochondrial proton gradient to generate heat, was significantly upregulated in white adipose tissue in these mice (34).

**Figure 1:**
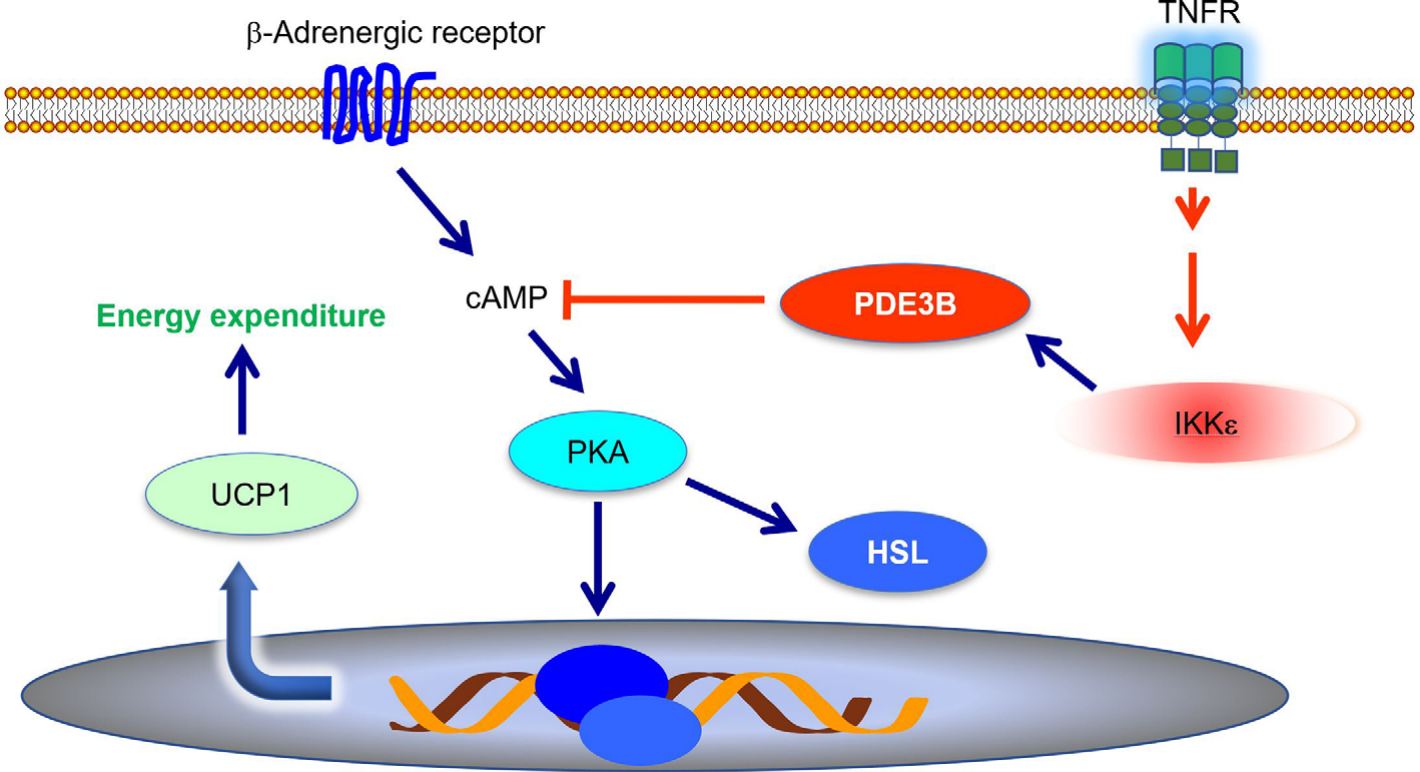
IKK*ϵ* inhibits adrenergic signaling to repress thermogenesis. IKK*ϵ* activity is induced by proinflammatory stimuli. Active IKK*ϵ* directly phosphorylates and activates PDE3B to reduce cAMP levels. Consequently, IKK*ϵ* inhibits cAMP-mediated adrenergic signaling pathway and represses energy expenditure in adipocytes. PDE3B, phosphodiesterase 3B; cAMP, cyclic AMP; PKA, protein kinase A; HSL, hormone sensitive lipase; UCP1, uncoupling protein 1.

Energy expenditure is largely controlled by sympathetic signals. Catecholamines induce *Ucp1* expression and increase thermogenesis in both brown and subcutaneous white fat (51, 52). During high fat diet-induced obesity, adipose tissue becomes resistant to catecholamines, resulting in decreased energy expenditure (9, 53–55). Mowers et al. demonstrated that IKK*ϵ* directly phosphorylates and activates phosphodiesterase 3B (PDE3B) to reduce intracellular cAMP levels and thus represses cAMP-mediated *β*-adrenergic signaling (55). *Ikke* knockout restored catecholamine sensitivity, leading to an upregulation of *Ucp1* expression and an increase of thermogenesis (34, 55, 56). Therefore, during obesity, the inflammation-induced expression of *Ikke* represses sympathetic signal and further promotes energy storage (**Figure 1**). IKK*ϵ* mediates the interaction between inflammatory and catecholamine signals, representing one example of how inflammation modulates metabolism in adipose tissue.

## TBK1

Although the role of TBK1 in NF*κ*B activation remains unclear, its function in the innate immune response has been well-recognized. In response to infection, pattern recognition receptors (PRRs) sense the pathogen-associated molecular patterns (PAMPs) on bacteria or viruses to activate TBK1-mediated signaling pathways (57, 58). Two major types of PRRs participate in this action. Toll-like receptors (TLRs), especially TLR3 and TLR4, are cell surface receptors that utilize adaptor proteins such as TIR-domain-containing adaptor-inducing interferon-β (TRIF) and Myeloid differentiation primary response 88 (MyD88). Ligands of TLRs, such as lipopolysaccharides (LPSs), bind to their receptors to induce the activation of TBK1. Retinoic acid-inducible gene I (RIG-I)-like receptors, NOD-like receptors (NLRs), and cytosolic DNA sensors are the PRRs in the cytoplasm (36, 59, 60). Cyclic-GMP-AMP (cGAMP) synthase (cGAS) is a cytosolic DNA sensor. cGAS utilizes cytosolic DNA to generate cGAMP, which in turn binds to the adaptor protein Stimulator of interferon genes (STING). Consequently, STING interacts with and activates TBK1 (61). Besides pathogen infection, proinflammatory cytokines such as tumor necrosis factor α (TNFα) also produces TBK1 activation (62, 63). Upon activation, TBK1 directly phosphorylates interferon regulatory factor 3 (IRF3) and IRF7 at multiple serine and threonine residues to induce their nuclear translocation (64–67). Consequently, these transcription factors upregulate the expression of type I interferon (*Ifna, Ifnb*) genes in the innate immune response. TBK1 is indispensable for the antiviral immune response (61, 68).

The activity of TBK1 is acutely controlled by phosphorylation on Ser^172^ within the kinase domain (63, 69, 70). However, the molecular mechanism by which this activating phosphorylation occurs is still unclear. Structural studies suggest that TBK1 undergoes multi-order oligomerization. While the kinase usually exists as a homodimer, the kinase domains face outward and are generally not capable of phosphorylation in this configuration (70). However, adapter proteins bring together these homodimers in larger heteromeric complexes, leading to Ser^172^ phosphorylation *via* transautophosphorylation (70). Moreover, recent investigations demonstrated that Unc-51 like autophagy activating kinase 1 (ULK1) can directly phosphorylate Ser^172^ (63). This is consistent with the observations that both ULK1 and TBK1 play essential roles in autophagy (71–75). TBK1 regulates autophagy *via* phosphorylating optineurin on Ser^177^ and SQSTM1/p62 on Ser^403^ to clear pathogen or damaged mitochondria (76, 77). Interestingly, the activation of NF*κ*B also upregulates the expression of *Sqstm1*/p62 to induce mitophagy in response to LPS (78, 79). These studies suggest that NF*κ*B and TBK1 may function synergistically to promote the clearance of damaged mitochondria and pathogens during infection.

Understanding the functions of TBK1 *in vivo* have been hampered by the lethality of global *Tbk1* knockout. Whole-body knockout of *Tbk1* leads to enhanced apoptotic liver degeneration and embryonic lethality at approximately E14.5 (80). In this regard, TBK1 directly phosphorylates receptor-interacting serine/threonine-protein kinase 1 (RIPK1) on Thr^189^ to prevent cell death. TBK1 deficiency substantially increases RIPK1-mediated cell death, resulting in embryonic lethality between embryonic day 13.5 and embryonic day 14.5 (81). In line with this finding, another study found that both TBK1 and IKK*ϵ* phosphorylate RIPK1 on multiple sites, including Thr^189^, to prevent TNF-induced cell death (62, 81). To conduct *in vivo* studies on the roles of TBK1 in inflammation, Marchlik et al., generated (*Tbk1* ^Δ/Δ^) mice expressing a TBK1 inactive mutant with the deletion of exon 2 (82). *Tbk1* ^Δ/Δ^ C57BL/6J mice were still embryonic lethal. However, *Tbk1* ^Δ/Δ^ 129S5 mice were fertile and viable, but born at a decreased Mendelian frequency. *Tbk1* ^Δ/Δ^ mice had increased mononuclear and granulomatous cell infiltration into multiple tissues, along with elevated circulating monocytes. This is consistent with another study reporting that *Tbk1* ^Δ/Δ^ mice die faster and in larger numbers in response to LPS (82).

### Regulation of the Crosstalk Between Metabolism and Inflammation by TBK1

**Figure 2:**
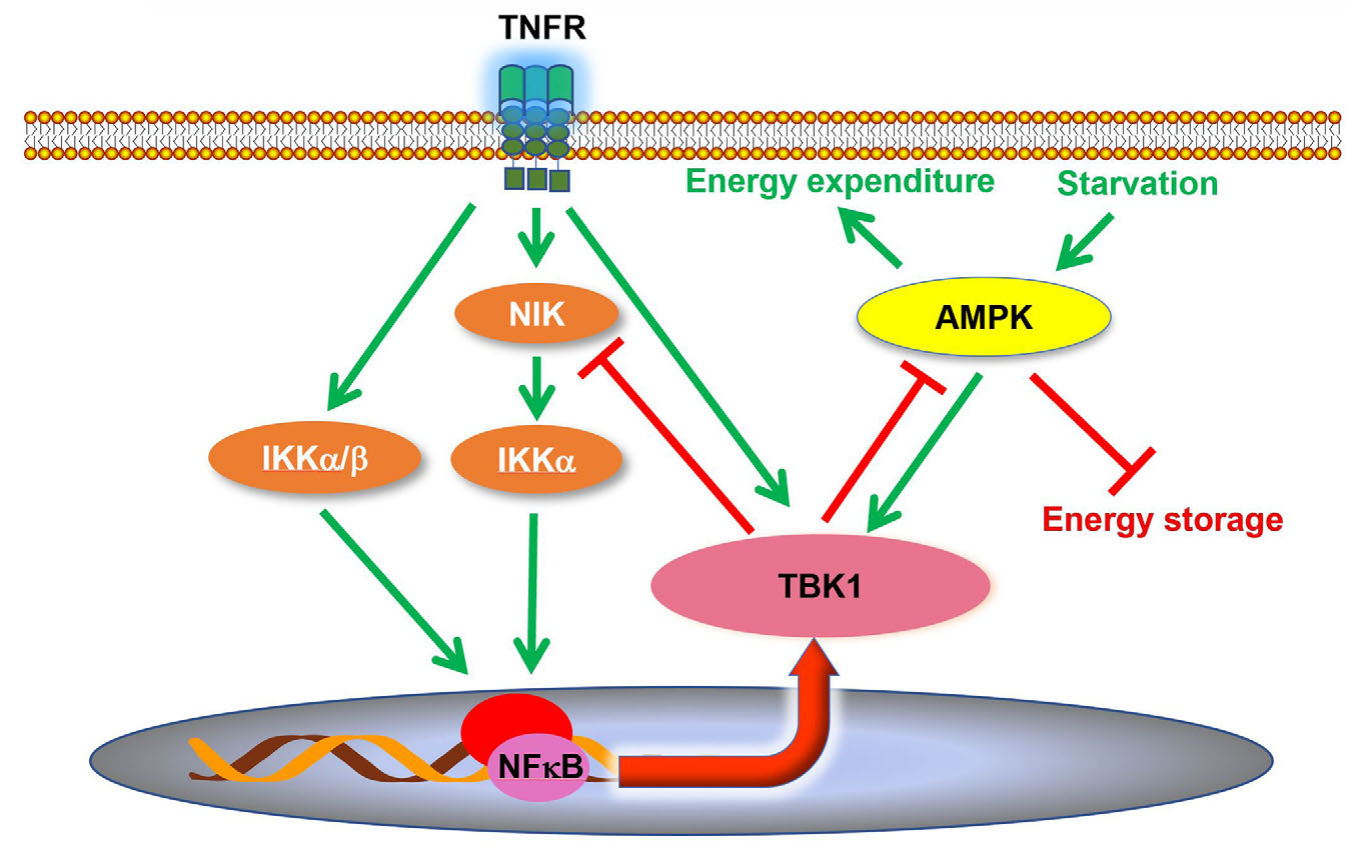
TBK1 regulates inflammation and energy metabolism in adipocytes. TBK1 activity is induced by proinflammatory stimuli and undernutrition. Although TBK1 is not directly involved in TNFα-induced activation of NF*κ*B, active TBK1 phosphorylates NIK to induce its degradation and thus attenuates atypical NF*κ*B pathway in a negative feedback loop. Moreover, TBK1 inhibits AMPK to repress energy expenditure in adipocytes. AMPK, AMP-activated protein kinase; NIK, NF*κ*B inducing kinase.

Although it was reported that TBK1 expression and activity are induced in adipose tissues during obesity and insulin resistance (34, 37, 63), the role of TBK1 in the pathogenesis of metabolic disease was unclear. A recent study revealed that TBK1 mediates crosstalk between inflammation and metabolism in adipose tissue (**Figure 2**) (63). During high fat diet-induced obesity, chronic inflammation leads to an increase of proinflammatory cytokines in the adipose tissue (9, 30, 83). Consequently, these cytokines, such as TNFα, produce the activation of TBK1 (63). At the same time, the inflammatory environment also results in enhanced NF*κ*B activity, resulting in an increase in *Tbk1* expression (34, 63). Thus, high fat diet feeding substantially induces TBK1 activity in the adipose tissue through both transcriptional and posttranslational regulation (34, 37, 63). Upon activation, TBK1 attenuates adipose tissue inflammation *via* repressing the atypical NF*κ*B pathway (63). In this pathway, the NF*κ*B-inducing kinase (NIK) phosphorylates Ser^176^ to activate IKK*α*, which largely resides as a homodimer (84). IKK*α* in turns phosphorylates the RelB (NF*κ*B2) precursor p100, resulting in the cleavage and maturation of RelB (85). Thus, NIK is responsible for activation of the atypical NF*κ*B pathway, which induces the expression of target genes, such as *Ccl2* (C-C motif chemokine ligand 2), to promote macrophage infiltration and inflammation (86–88). Interestingly, TBK1 directly phosphorylates NIK, leading to its degradation (62, 63). *Tbk1* knockout causes hyperactivation of the atypical NF*κ*B pathway and exacerbates macrophage infiltration and inflammation in adipose tissue of obese mice (63). Moreover, the loss of TBK1 in adipocytes attenuates HFD-induced obesity *via* increasing mitochondrial biogenesis and energy expenditure. TBK1 inhibits AMP-activated protein kinase (AMPK) by catalyzing phosphorylation on inhibitory sites in AMPK*α* subunit, Ser^459^ and Ser^476^. *Tbk1* knockout thus ameliorates AMPK repression in adipose tissues of high fat diet-fed mice (63), revealing that TBK1 mediates crosstalk from inflammation to energy metabolism. The inflammation-induced TBK1 activity produced during obesity represses energy expenditure and promotes anabolism, which further enhances obesity through a feedforward loop.

In addition to inflammation-induced TBK1 activation, it has also been reported that TBK1 Ser^172^ phosphorylation is induced in adipocytes during glucose deprivation, which creates an energy shortage condition (63). Thus, TBK1 is activated not only during overnutrition, but also during undernutrition. Mechanistically, energy shortage leads to an increase of AMP/ATP ratio, which in turns activates AMPK. AMPK directly phosphorylates ULK1 at multiple residues to induce its activity (89, 90). ULK1 is able to phosphorylate Ser^172^ to activate TBK1 (63). Similar observations on AMPK-dependent TBK1 activation have been reported in myotubes and Hela cells as well (91). Furthermore, prolonged fasting induced *Tbk1* expression in different depots of white adipose tissues (63). However, the molecular mechanism of this transcriptional regulation is still unknown. Studies on animal models and human subjects reported that fasting or undernutrition leads to a reduction of basal metabolic rate and energy expenditure (92, 93). Given the effects of TBK1 on energy metabolism, fasting likely activates a TBK1-mediated feedback loop to repress energy expenditure in response to undernutrition. The activation of TBK1 could be a protective mechanism to attenuate the loss of body weight during fasting. Moreover, reduced caloric intake has been demonstrated to attenuate adipose tissue inflammation in obesity (94–97). The anti-inflammatory function of TBK1 at least partially contributes to this effect and mediates crosstalk from undernutrition to inflammation.

In summary, TBK1 plays a central role in the regulation of both inflammation and energy metabolism in adipose tissue. It is activated during both overnutrition and undernutrition and mediates a negative feedback loop to repress inflammation and energy expenditure under certain conditions (63). More importantly, TBK1 is responsible for the bidirectional crosstalk between energy metabolism and inflammation. The deficiency of TBK1 in adipocytes leads to the attenuation of high fat diet-induced obesity, but the exaggeration of adipose tissue inflammation (63), indicating a loss of the positive correlation between adiposity and adipose tissue inflammation.

Furthermore, in response to proinflammatory stimuli, TBK1 has been shown to affect metabolic reprogramming in different cell types. Upon the activation of TLRs, active TBK1 was recruited to the myddosome and thus promotes glycolysis in macrophages (98). Another two studies also reported that TBK1 activation mediates TLR ligand-induced glycolytic reprogramming (99, 100). The rapid induction of glycolysis is critical for the production of succinate and inflammatory cytokines in the immune response (99). These findings demonstrate another TBK1-mediated pathway that regulates the crosstalk between inflammation and metabolism. However, further studies are needed to compare the cell type specific roles of TBK1.

### Inhibition of TBK1 and IKK*ϵ* in Metabolic Diseases

Insights into the critical roles of the noncanonical IKKs in the pathogenesis of obesity and insulin resistance led to a screen of chemical inhibitors, identifying amlexanox as an inhibitor for both TBK1 and IKK*ϵ* (37). Daily gavage of amlexanox in obese mice prevents genetic and high fat diet-induced obesity. The inhibition of weight gain by amlexanox is reversible after withdrawal of the drug. Amlexanox improved insulin sensitivity, reduced adipose tissue inflammation, increased energy expenditure, and attenuated hepatic steatosis in these obese animal models (37). Considering the phenotypes observed in *Ikke* knockout mice and adipose *Tbk1* knockout mice, the beneficial effects of amlexanox is likely the combined outcomes from the inhibition of both kinases. The inhibition of IKK*ϵ* increases cAMP and catecholamine sensitivity to upregulate thermogenesis and attenuates adipose tissue inflammation (34). On the other hand, loss of TBK1 activity de-represses AMPK to increase mitochondrial biogenesis and other catabolic functions (63). The TBK1 deficiency-induced adipose tissue inflammation is likely compensated by the anti-inflammatory effects of IKK*ϵ* inhibition.

In a proof-of-concept randomized, double-blinded clinical study, 42 obese and diabetic patients received placebo or amlexanox treatment for 12 weeks. Amlexanox significantly reduced hemoglobin A1c levels (101), indicating an improvement of glucose metabolism. Further study found that patients with higher serum C-reactive protein (CRP) levels and higher adipose tissue inflammation were more responsive to the drug. In the responder group, amlexanox improved insulin sensitivity and hepatic steatosis. The expression of thermogenic genes, including *Ucp1*, *Dio2* and *Fgf21*, was upregulated by the treatment as well in these patients. Within the responders, a transient increase of serum Interleukin 6 (IL-6) within 2–4 weeks of amlexanox treatment was reported (101). This observation is consistent with a previous mouse study showing that amlexanox upregulated *Il6* expression and secretion *via* cAMP/Mitogen-activated protein kinase (MAPK) p38 pathway in inguinal white adipose tissue. The increase of circulating IL-6 activates Signal transducer and activator of transcription 3 (STAT3) in the liver to inhibit the expression of the gluconeogenic gene Glucose-6-phosphatase (*G6pc*). As a result, amlexanox represses hepatic glucose output and thus improves glucose tolerance (102).

## Concluding Remarks

Although the causal relationship between inflammation and obesity-associated metabolic disorders remains uncertain, there is little doubt that adipose tissue inflammation correlates well with the occurrence of insulin resistance and type 2 diabetes (16, 17, 19, 20). The crosstalk between inflammation and metabolism in adipose tissue plays a critical role in the pathogenesis of metabolic diseases. Overnutrition causes metabolic stress, which induces the initiation of inflammation to restore the metabolic homeostasis (9). The activation of proinflammatory signaling pathways attenuates insulin responsive signals to prevent further energy storage in adipocytes (103, 104). Both of these effects are the physiological/adaptive responses to overnutrition. However, along the progression of obesity, sustained inflammation causes a shift of homeostatic setpoints, leading to hyperglycemia, hyperinsulinemia, and reduced energy expenditure (9). At this stage, the inflammation causes a pathological/maladaptive response that further exaggerates obesity and obesity-associated metabolic disorders. Therefore, sustained inflammation results in a transition from an adaptive response to a maladaptive response that accelerates the progression of metabolic disorders.

NF*κ*B signals mediate inflammatory responses and interact with metabolic pathways in adipose tissue (33, 105, 106). The activities of non-canonical IKKs, TBK1, and IKK*ϵ* are induced during inflammation (34, 37, 63). TBK1 represses energy expenditure *via* inhibiting AMPK, while IKK*ϵ* desensitizes sympathetic signals (34, 55, 63). The activation of these kinases exacerbates adiposity accumulation and promotes obesity. A recent study reported that escaped mitochondrial DNA activates TBK1 and IKK*ϵ* to repress energy expenditure during metabolic stress (56). Amlexanox, a drug with outstanding safety record, was identified as an inhibitor of TBK1 and IKK*ϵ*. Thus far, multiple studies on both experimental mouse models and human subjects suggest its potential as a new treatment for metabolic diseases (37, 101).

In addition to modulating metabolic pathways in adipocytes, metabolic and inflammatory signals interact at systemic level in other cell types. Metabolic stress has the potential to increase the production of adipokines, including leptin, adiponection, and others (28–31). It has been reported that leptin induces inflammation, while adiponectin attenuates inflammation (107–110). Moreover, metabolic status could affect the functions of immune cells. Caloric restriction has exhibited systemic anti-inflammatory effects, along with attenuated terminal differentiation of immune cells (111). Given the energy sensing properties of AMPK, the AMPK–ULK1–TBK1 axis may also function in immune cells to mediate anti-inflammatory effects. Nonetheless, the precise roles of adipose tissue inflammation in the progression of obesity and obesity-associated insulin resistance remains unclear. Indeed, more efforts are needed to understand the systemic interactions between immune and metabolic responses, which are essential for the maintenance of homeostasis.

## Author Contributions

PZ prepared the manuscript. AS reviewed and edited the manuscript. All authors contributed to the article and approved the submitted version.

## Funding

The study is supported by NIH P30DK063491, R01DK076906, R01DK117551, R01DK122804, and R01DK125820 to AS.; NIH K99HL143277 to PZ.

## Conflict of Interest

AS is a founder of Elgia Therapeutics.

The remaining author declares that the research was conducted in the absence of any commercial or financial relationships that could be construed as a potential conflict of interest.
